# The Global Epidemic of the Metabolic Syndrome

**DOI:** 10.1007/s11906-018-0812-z

**Published:** 2018-02-26

**Authors:** Mohammad G. Saklayen

**Affiliations:** 0000 0004 1936 7937 grid.268333.f V.A. Medical Center, Wright State University Boonshoft School of Medicine, 4100 West Third St, Dayton, OH 45428 USA

**Keywords:** Metabolic syndrome, Insulin resistance, Prediabetes, Obesity, Abdominal obesity, Waist-hip ratio, Healthy herbs, Leisure time physical activity (LTPA)

## Abstract

Metabolic syndrome, variously known also as syndrome X, insulin resistance, etc., is defined by WHO as a pathologic condition characterized by abdominal obesity, insulin resistance, hypertension, and hyperlipidemia. Though there is some variation in the definition by other health care organization, the differences are minor. With the successful conquest of communicable infectious diseases in most of the world, this new non-communicable disease (NCD) has become the major health hazard of modern world. Though it started in the Western world, with the spread of the Western lifestyle across the globe, it has become now a truly global problem. The prevalence of the metabolic syndrome is often more in the urban population of some developing countries than in its Western counterparts. The two basic forces spreading this malady are the increase in consumption of high calorie-low fiber fast food and the decrease in physical activity due to mechanized transportations and sedentary form of leisure time activities. The syndrome feeds into the spread of the diseases like type 2 diabetes, coronary diseases, stroke, and other disabilities. The total cost of the malady including the cost of health care and loss of potential economic activity is in trillions. The present trend is not sustainable unless a magic cure is found (unlikely) or concerted global/governmental/societal efforts are made to change the lifestyle that is promoting it. There are certainly some elements in the causation of the metabolic syndrome that cannot be changed but many are amenable for corrections and curtailments. For example, better urban planning to encourage active lifestyle, subsidizing consumption of whole grains and possible taxing high calorie snacks, restricting media advertisement of unhealthy food, etc. Revitalizing old fashion healthier lifestyle, promoting old-fashioned foods using healthy herbs rather than oil and sugar, and educating people about choosing healthy/wholesome food over junks are among the steps that can be considered.

## Introduction

With the successful conquest of many of the old infectious diseases in the world, non-communicable diseases (NCD) have become the major cause of morbidity and mortality not only in the developed world but also in the underdeveloped countries. Among all these NCD, metabolic syndrome had been the real scourge globally.

Metabolic syndrome (MetS), also variously known as syndrome X, Insulin resistance, etc. in the literature, is really not a single disease but a constellation of cardiovascular disease risk factors and had been defined slightly differently by various organizations. Three most popular definitions [1] used for surveys and health care plan are:

### WHO 1999:

Presence of insulin resistance or glucose > 6.1 mmol/L (110 mg/dl), 2 h glucose > 7.8 mmol (140 mg/dl) (required) along with any two or more of the following:

1. HDL cholesterol < 0.9 mmol/L (35 mg/dl) in men, < 1.0 mmol/L (40 mg/dl) in women
2. Triglycerides > 1.7 mmol/L (150 mg/dl)
3. Waist/hip ratio > 0.9 (men) or > 0.85 (women) or BMI > 30 kg/m^2^
4. Blood pressure > 140/90 mmHg

### NCEP (National Cholesterol Education Program) ATP3 2005:

Presence of any three or more of the following:

1. Blood glucose greater than 5.6 mmol/L (100 mg/dl) or drug treatment for elevated blood glucose
2. HDL cholesterol < 1.0 mmol/L (40 mg/dl) in men, < 1.3 mmol/L (50 mg/dl) in women or drug treatment for low HDL-C
3. Blood triglycerides > 1.7 mmol/L (150 mg/dl) or drug treatment for elevated triglycerides
4. Waist > 102 cm (men) or > 88 cm (women)
5. Blood pressure > 130/85 mmHg or drug treatment for hypertension

### IDF (International Diabetes Federation) 2006:

Waist > 94 cm (men) or > 80 cm (women) along with the presence of two or more of the following:

1. Blood glucose greater than 5.6 mmol/L (100 mg/dl) or diagnosed diabetes
2. HDL cholesterol < 1.0 mmol/L (40 mg/dl) in men, < 1.3 mmol/L (50 mg/dl) in women or drug treatment for low HDL-C
3. Blood triglycerides > 1.7 mmol/L (150 mg/dl) or drug treatment for elevated triglycerides
4. Blood pressure > 130/85 mmHg or drug treatment for hypertension
  Note: *NCEP and IDF definition are very similar except in the waist parameter of 102 vs. 94 cm in men and 88 vs. 80 cm in women.*

Other organizations like the American Association of Clinical Endocrinologist (AACE) 2003 and the European Group for the Study of Insulin Resistance (EGIR) used slightly different definitions but they are not as commonly used.

## Incidence and Prevalence of Metabolic Syndrome

The incidence of metabolic syndrome often parallels the incidence of obesity and incidence of type 2 diabetes (one of the outcome of MetS). According to NHNES data, during 1988–2010, average BMI in USA increased by 0.37% per year in both men and women and waist circumference (WC) increased by 0.37 and 0.27% per year in women, respectively. According to CDC data published in 2017, about 30.2 million adults aged 18 years or older or 12.2% of USA adults had type 2 diabetes (T2DM). *One quarter of these people (23.8%) were not aware of having diabetes.* Incidence of T2DM increased with age, reaching a high of 25.2% among US seniors (65 years or older). Prevalence of prediabetes or MetS was about three times more. So, about one third of US adults have metabolic syndrome [2].

Incidence of T2DM was even higher among certain ethnic groups—15% among American Indians but lower among Chinese Americans − 4.3%. South Asian Americans had a very high prevalence of metabolic syndrome along with higher incidence of abdominal obesity [3].

In China, between 1992 and 2002, the prevalence of overweight and obesity increased from 14.6 to 21.8%—based on WHO criterion. Using the Chinese obesity definition, with lower BMI cutoff, the increase was from 20 to 29%. The incidence of MetS increased from 8 to 10.6% in urban areas and 4.9 to 5.3% in rural areas. Assuming the same rate of increase, the prevalence of MetS in China in 2017 would be about 15.5% [4].

According to global survey of obesity in 195 countries, done in 2015, 604 million adults and 108 million children were obese. Since 1980, prevalence of obesity doubled in 73 countries and increased in most other countries. Of even greater concern was that the rate of increase was even higher in childhood obesity [5].

According to this survey, obesity is no longer a disease of affluence. The highest increase in prevalence of obesity in young men (25–29 years) occurred in countries with low socio-economic index (SDI). In the last three decades, the prevalence went from 1.1% in 1980 to 3.85 in 2015. Between 1990 and 2015, global rate of death related to high BMI increased by 28.3%. *Obesity also contributed to 120 million disability-adjusted life-years.* The highest percentage change in age-standardized BMI related deaths and disability-adjusted life-years occurred in Bangladesh—*one of the poorest country and the country where the present author comes from.* On the other hand, age-standardized BMI-related morbidity and mortality decreased by 37.2 and 43.7%, respectively, in Turkey.

Obesity, however, is not always synonymous with MetS. There are so-called metabolically healthy obese (MHO) individuals who have high level of insulin sensitivity and do not have hypertension and hyperlipidemia and other features of MetS. Epidemiological survey suggests that MHO may account for a significant percentage of obese population [6]. According to CoLaus study—a single center cross-sectional study including a random sample of 6188 extensively phenotyped, Caucasian subjects aged 35–70 years living in Lausanne, Switzerland—the prevalence of overweight, obesity, hypertension, hyperlipidemia, diabetes, and microalbuminuria was 36.6, 15.7, 36.7, 34.2, 6.6, and 6.3%, respectively, and in this population (unlike in Middle east) in all categories, prevalence was higher in men than women. Prevalence increased with age which is true for all other population study [7].

According to IDF diabetes atlas [8], global prevalence of diabetes is 8.8% (415 m) as of 2015 and is expected to increase to 10.4% (642 m) by 2040. The highest prevalence of diabetes was in North American and Caribbean region (11.5%). Over half of all people with diabetes were living in Southeast Asia and Western Pacific region. Prevalence is still relatively low in Africa region. But in next 25 years, some of the highest growth rate in diabetes is expected to be in sub-Saharan Africa and Middle East/North Africa (141 and 104%, respectively).

We do not have similar global data on metabolic syndrome—which is harder to measure, but since MetS is about three times more common than diabetes, the global prevalence can be estimated to be about one quarter of the world population. *In other words, over a billion people in the world are now affected with metabolic syndrome.*

The prevalence estimates vary, based on the criteria used for the definition of MetS. For example, a national survey in Iran in 2007 showed prevalence of MetS was about 34.7% based on ATP III criteria, 37.4% based on IDF definition, and 41.6% based on ATP III/AHA/NHLBI criteria. In another Middle Eastern country, Tunisia, prevalence was 45.5% based on IDF criteria but 24.3% based on ATP III criteria. But in all the Middle Eastern countries, prevalence was much higher among women than men [9].

## Biology of the Metabolic Syndrome

During the last three decades, while prevalence of MetS increased, our understanding of the biology of the malady also increased. All fats are no longer considered the same. Adipocytes are now categorized as *white adipocytes*, *brown adipocytes*, and *beige adipocytes*. The brown and beige adipocytes are morphologically and functionally different than white adipocytes. These cells have more mitochondria in the cytoplasm enriched with more uncoupling protein 1 (UCP1) and able to produce more thermogenesis [10].

Rather than being an inert energy storage depot, adipocytes are now known to be metabolically active, secreting over a dozen of hormones affecting the appetite, satiety, and energy metabolism of the body. While first known adipocyte hormone leptin suppresses appetite and genetic absence of which causes massive obesity, other hormones like adiponectin have just the opposite effect [11]. Adiponectin increases insulin sensitivity, as well as pancreatic beta cell survival and functionality. Overexpression of adiponectin had profound positive effects on adipose tissue, e.g., increase in mitochondrial density, decrease in the size of adipocytes, and transcriptional upregulations of factors related to efficient esterification of free fatty acids. Recently, adiponectin was found to be protective against the metabolic syndrome in a polycystic ovary syndrome mouse model [12].

The macrophages residing in the fat tissue are a major player in energy metabolism. Pro-inflammatory (M1) macrophages promote hepatic steatosis and adipogenesis, while anti-inflammatory macrophages (M2) do the opposite. Macrophage-restricted deficiency of transcriptional regulator methyl-CpG-binding protein 2 (MECP2) also caused hepatic steatosis and obesity phenotype in a mice model [13].

In a recent study, Chug et al. reported that M1 macrophage retention in adipose tissue is mediated by *alphabeta1* integrin. The process is also dependent on expression of VCAM1 which encodes vascular cell adhesion molecules—the counter receptor for *alpha-beta1* integrin. The retention and activation of M1 macrophages in fat tissue inhibit adipocyte UCP1 expression and beige adipocytes. The role of *alphabeta*1 integrin in obesity is particularly important since there is already an FDA-approved drug *Natalizumab*, a monoclonal antibody that blocks this integrin, used in the treatment of multiple sclerosis.

Endoplasmic reticulum (ER) stress is another important factor in the pathogenesis of metabolic syndrome. In mice experiment, Shan et al. showed that high fat diet (HFD)-induced ER stress is dependent on inositol-requiring enzyme 1 alpha (*IRE1alpha*) activity. Deficiency of *IRE1alpha* prevented obesity, insulin resistance, and hepatic steatosis induced by HFD. *IRE1alpha* also suppresses the M2 macrophages in in adipose tissue [14].

One of the phenotypic features of MHO is their strong skeletal built. Underlying physiology of this is now better understood. An *osteoblast*-derived hormone Lipocalin2 (LCN2) maintains glucose homeostasis by inducing insulin secretion and insulin sensitivity. LCN2 also inhibits food intake by suppressing melanocortin 4 receptor (MC4R) in the paraventricular and ventromedial neurons of the hypothalamus and activates MC4R-dependent anorexigenic pathway [15].

Thyroid hormones along with beta-adrenergic hormones are well-known factors regulating energy metabolism. Miao et al. recently described the interaction of thyroid hormones and liver x receptor (LXR) which serves as repressors of UCP1 in classic brown adipocytes. Depletion of LXR in mice activated *thyroid stimulating hormone (TSH)-releasing hormone (TRH)-positive* neurons in the hypothalamus and stimulated TSH from the pituitary gland leading to increased secretion of thyroid hormone. Also, decreased expressions of perinatal deiodinase 2 in hepatocytes greatly reduce the susceptibility to diet induced steatosis and obesity [16].

Gut microbiome has now become an important part of human biology in health and diseases. Healthy microbiome prevents many cardiovascular diseases as well as MeTS/diabetes. Everard et al. isolated one specific gut microbe *Akkermansia muciniphila*, a mucin-degrading bacterium that resides in the mucus layer of gut epithelium. Feeding mice with perbiotic (oligofructose) normalized *A. muciniphila* abundance (increased by 100-fold) and corrected the metabolic syndrome in the obese mice. *A. muciniphila* administration increased the intestinal level of endocannabinoids that control the inflammation, gut barrier, and gut peptide secretion. This group now reported that use of membrane protein from *A. muciniphila* improves metabolic syndrome in mice [17, 18].

In addition to these, other biologic mechanisms of MetS, recently discovered, have potential therapeutic implication. Kopec et al. showed that thrombin promotes diet-induced obesity through fibrin-driven inflammation and that treatment with thrombin inhibitor drug dabigatran—already in use for treatment of DVT—limited HFD-induced obesity and MetS [19]. Maternal vitamin D supplementation in mice reduced MetS in the offspring [20]. Recently, Cox et al reported a specially designed *PPARdelta* agonist which when used in mice reduced the metabolic syndrome [21•].

In all epidemiologic studies, prevalence of MetS increases with age. This is not surprising since there are many commonalities in biochemical changes of aging process and metabolic syndrome/diabetes [22].

## Genetics and Epigenetics of Obesity/MetS

While there are some known genes associated with obesity and MetS, the epidemic growth of the malady in a short period makes genetic predisposition a minor component. A genome-wide association study and Metabochip meta-analysis of BMI done in 339,224 individuals identified 97 BMI-associated loci, 56 of which were novel. The 97 loci accounted for ~ 2.7% of BMI variation and common variation accounted for > 20% BMI variation [23].

However, epigenetics seems to have a bigger role in promoting MetS. Parenteral obesity may cause obesity in the offspring through epigenetic changes in the spermatozoa or oocytes or more commonly in utero environment. Children born to obese mother or father who underwent bariatric surgery prior to conception of the children are less prone to obesity/MetS than children born before bariatric surgery [24].

Epidemiological studies have shown strong association between intrauterine nutrition, patterns of postnatal nutrition, and growth and metabolic syndrome in adults. Mothers exposed to the Dutch famine of 1944/45 during the first two trimesters of the pregnancy had babies with low birth weights (LBW), but these babies had higher incidence of obesity in adult life. LBW infants, who had rapid catch up growth as infant, had the highest risk of developing obesity and MetS in adult life. A similar phenomenon was observed in China after the famine there in 1959–1961. Mechanistically, this phenomenon seems to occur via decreased DNA methylation of the imprinted IGF2 gene in the offspring and hypermethylation of two obesity-related genes—leptin and TNF. In rodent models of maternal under- or over-nutrition and newborn-specific nutritional modifications, epigenetic changes were seen that affected growth factors, adipogenesis, appetite control, and glucose homeostasis in the offspring [25].This phenomenon is of particular concern and the high incidence of obesity and MetS in the newly developing countries may be explained by this mechanism.

## Prevention

### Exercise

Physical activity (PA) and exercise are key components of energy expenditure and energy balance. But the benefit of exercise in preventing metabolic syndrome goes beyond the immediate benefit of caloric expenditure [26]. With chronic exercise or increased PA, there are structural changes in the muscles, increase in number of mitochondria in fiber, secretion of metabolically beneficial hormone like *Irisin* with reversal of muscle insulin resistance, and reduction in postprandial hepatic lipogenesis [27].

However, according to NHANES data during the period of 1988 and 2010, when the average BMI and WC increased in US adults, the proportion of adults who reported NO leisure lime physical activity (LTPA) increased from 19.1 to 51.7% in women and from 11.4 to 43.5% in men. According to this study, average caloric intake did not change during this time and BMI and WC trends were associated with LTPA level and not caloric intake [28].

According to recent CDC data, 40.8% of adults in USA were physically inactive (defined as < 10 min a week moderate or vigorous activity in each of the PA categories of work, leisure time, and transportation). While we do not have easy access to such physical activity data in the developing countries, it can be assumed that with the acceptance of Western lifestyle, increased use of automobiles and more time spent indoor watching TV or playing video games, the data trend will be very similar. Increased sedentary lifestyle is a major driving force for increased prevalence of MetS [29].

### Diet

Predimed and other studies provided evidence supporting a beneficial role of traditional Mediterranean diet in preventing diabetes and metabolic syndrome. In the Predimed study, it was also observed that just an ounce of *extra virgin olive oil* (EVOO) given as supplement to usual Western type diet reduced the incidence of MetS and hypertension [58].

Several dietary factors are known to prevent MetS. Notable among these *are olive oil*, *capsaicin*, *luteolin*, *curcumin*, *cinnamon*, *rosemary*, etc.

A systemic review on the effects of dietary polyphenols on metabolic syndrome done recently showed that, at relatively high doses, many polyphenols favorably influence different features of metabolic syndrome. Soy isoflavone, citrus products, hesperidin, and quercetin improved lipid metabolism, and cocoa supplement improved high blood pressure and blood glucose. Green tea significantly reduced BMI and waist circumference and improved lipid metabolism [30].

Epidemiologic data show that consumption of foods high in capsaicin is associated with lower prevalence of obesity/MetS. Capsaicin present in hot pepper works on the TRPV1 and PPAR alpha receptors and reduces metabolic dysregulation in obese/diabetic mice by enhancing expression of adiponectin and its receptor [31, 32].

Cheng et al. recently reported that low-calorie, low-protein, and low-carb but high fat fasting mimicking ketogenic diet for 4 days increased the generation of pancreatic beta cells and reversed both T1D and T2D phenotypes in mouse models [33•].

(In mice, a 4-day fasting mimicking diet (FMD) induces a stepwise expression of Sox17 and Pdx-1, followed by Ngn3-driven generation of insulin-producing β cells, resembling that observed during pancreatic development.) FMD cycles restore insulin secretion and glucose homeostasis in both type 2 and type 1 diabetes mouse models. In human type 1 diabetes pancreatic islets, fasting conditions reduce PKA and mTOR activity and induce Sox2 and Ngn3 (Neurogenin 3) expression and insulin production.

Liu et al. recently reported in *Nature* that blocking follicle-stimulating hormone (FSH) induces thermogenic adipose tissue and reduces body fat [34]. A non-classic eicosanoid Lipoxin A4 also has been shown to attenuate obesity-induced adipose inflammation and associated liver and kidney disease [35].

Of the traditional approved drug, the most effective medicine in preventing MetS is metformin which is a unique anti-diabetic medicine whose mechanism of action is not fully understood yet but closely mimics the effect of exercise [36, 37•, 38]. There are many functional foods—mostly herbs that are effective in preventing metabolic syndrome to some extent.

Carnosic acid is a major bioactive component in Rosemary extract and it is found to ameliorate HFD-induced obesity and metabolic syndrome in mice [39]. Recently, Indian investigators showed that a single supplement intervention of 3 g cinnamon for 16 weeks resulted in significant improvements in all components of metabolic syndrome in a North Indian cohort [40]. Another herbal product that has shown promise in combating MetS is Ashwagandha [41, 42].

Galantamine is a centrally active acetyl cholinesterase inhibitor with anti-inflammatory properties and is FDA-approved for treatment of dementia. According to a recent RCT done in Brazil, daily intake of 8–16 mg of galantamine orally for 4 weeks reduced plasma levels of TNF and leptin and increased level of adiponectin and IL-10 and significantly lowered plasma insulin and insulin HOMA-IR [43]. If these results are confirmed, galantamine may be a useful drug for alleviation of metabolic syndrome. Another herbal product Celastrol has shown promise in combating obesity and MetS in mice model [44]. Kefir—a dairy-based drink popular in some culture—is now showing up in Western supermarket because of its effect in preventing metabolic syndrome [45].

But knowledge of special diet and use of different herbs/functional foods can only go so far. To really stop and control the epidemic of MetS, a societal/governmental/global approach is urgently needed.

Civil societies, outside of the strict medical community, need to help disseminate the awareness of the huge impact of MetS on the overall health in the global community. Civil society can help disseminate the awareness and relevant issues to the common citizenry through discussion, debates/lectures, and effective use of mass media. Rather than viewing the obesity and leanness as an anthropometric and fashion issue, attention should be directed to the associated health issue with acknowledgement that all obese persons are not metabolically unfit and all lean people are not metabolically fit [46•].

Producers of literatures on health should try to popularize the waist/hip ratio concept as better matrix of metabolic syndrome than BMI [47]. Building a culture of physical fitness and healthy diet from cradle to the grave should be the societal goal [48•].

Newer technology can be used to educate and create awareness. Use of Facebook and electronic apps may be used in promoting healthy lifestyle. Newer technology is also needed in creating better food quality supply. We know that eating whole grain product is less likely to cause MetS than refined carbohydrate [49]. A simple improvement in machines used in milling the rice so that the husk with fibers are not totally removed as in “white” rice, or “white” flour, can make a big difference in the quality of the primary carbohydrate consumed by millions.

Governmental involvements at municipal levels to country level are very important. Sometime, a minor change in law can accomplish much more than many lectures and good advice. The small tax imposed on sugary drinks in New York City, Philadelphia, or at the country level like in Mexico was effective in lowering consumption of sugary drinks [50]. But laws need to be carefully crafted and be a part of total societal resolve to counter the problem obesity/MetS. Otherwise, it can be a dismal failure. One example is mandatory labeling of fat content in some food items in the USA. The result was many food items with low or no fat but higher sugar content without change in total calorie content. Also, scapegoating fructose as causing metabolic syndrome without giving similar restriction on overall sugar/calorie consumption did not prevent the spread of obesity in the USA [51•].

Government can help create life-long health impact by ensuring good minimal perinatal care and promotion of breast feeding for every newborn. It can also introduce good cooking lesson and basic nutritional knowledge in secondary schools. Generations of young people are now entering adulthood without knowing how to cook and thereby depending on fast foods for survival. If these young men and women discover the joy of cooking, promoting healthy diet among them will be easier.

Governments can also ensure creation of more parks and pedestrian walkways during urban development plan. One Latin American city once banned its citizens from using any automobile one Sunday a month. The result was astonishing. Suddenly, there were swarms of kids and adults in the street playing football or other games and no one was driving. It was magical.

And lastly, governments can lead in educating the health care provider about the enormous economic cost of the MetS epidemic and mandate some incentive for preventive care. But medical doctors are not well suited for preventive care and cadres of barefoot doctors may need to be used for this purpose. Since in many countries vaccination programs are used by such providers, they can be retrained now for the prevention of this new epidemic.

WHO or similar organizations can work with country level governments to make country-specific plan to confront the epidemic, keeping in mind the cultural, economic, and educational matrix of individual country [52•]. While it is a global epidemic, there is wide variation in the prevalence within similar socio-economic strata of countries. WHO can study what is right with the countries which are successful in controlling MetS and what is wrong elsewhere [53, 54].

And lastly, health care providers need to take leadership position in directing the society towards a healthy community that are not only free of infectious disease but are physically fit and enjoying disease-free long life. Most practicing clinicians do not talk to the patients about good eating habits and need for adequate physical activity. But few words of such reminders and encouragements during routine visit can go a long way in changing people’s habit.

Lastly, many of the MetS are iatrogenic due to wide use of some drugs especially the new generation of anti-depression and anti-psychotic meds [55•]. Many HIV drugs are also obesogenic. Patients may need to be particularly warned about these side effects and taught some preventive measure early on. Preventive use of metformin and similar drugs may be useful in such patients and others who are at high risk for developing MetS.

## Conclusion

The metabolic syndrome is a complex pathophysiologic state that originates primarily from an imbalance of calorie intake and energy expenditure but also affected by genetic/epigenetic make up of individual, predominance of sedentary lifestyle over physical activity, and other factors like quality and composition of food and composition of gut microbes. No single remedy can be prescribed for its eradication or even curtailment.

The epidemic did not happen suddenly and it cannot be controlled quickly, but if there is a societal will, it can be done. As in the control of other epidemics, education of the population about the health hazard of the metabolic syndrome will be very important. Since metabolic syndrome is a complex medical diagnosis requiring blood tests, simple anthropometric definition that can be easily used and accepted by populace is necessary. While exact measure to be taken can be debated, promoting the danger of increase in abdominal girth or waist/hip ratio can be a starting point. A slogan like, if your belt size increases by a notch, danger is lurking inside the belly can be promoted. Or “sweets are for weak-lings, hot peppers are for the machos.”

Targeting the younger population to prevent MetS before it happens is a better plan than trying to cure it after it happens. While a dedicated group of public health planner can create a comprehensive plan for a given society or a given community, here are some suggestions:

1. Cooking skills should be taught to the students in high school/college. Without learning to cook healthy yet enjoyable food, the dependency on fast food cannot be controlled. Many commercial ventures in the USA that are helping young couples to cook are a good start but they are expensive and not widely adoptable.
2. Governments need to stop subsidizing unhealthy food (farm support bill in the USA).
3. Before embarking on any large-scale change, planners need to do pilot studies to see what works and what does not.
4. Increase investment in finding pharmaceutical assistance by developing drugs like metformin.
5. Understand the biology even better. Recently, three publications in *Nature* medicine reported the biological importance of GDF15 and its receptor in maintaining energy balance [56, 57•]. These and other recent understanding of energy metabolism need to be translated in practical application in humans.
6. The present epidemic of MetS is also related to the wide acceptance of predominantly Western capitalistic economic development model. A better, more humane economic development plan can address the inherent health hazards of this capitalistic model where profits supercede long-term effects on the society. Better understanding of socio-economic plans that promote conditions favoring the MetS epidemic can help choose alternate development plan.
